# Historical comparative genomics to track the evolution of fungal pathogens: a proof of concept

**DOI:** 10.1186/s12864-025-12472-2

**Published:** 2026-01-12

**Authors:** Edgar L. Y. Wong, Joy Lyu, Olivia Tjahjono, Joris A. Alkemade, Alan G. Buddie, Matthew J. Ryan, Timothy G. Barraclough

**Affiliations:** 1 Department of Biology, University of Oxford, Oxford, UK; 2 Senckenberg Biodiversity and Climate Research Centre, Frankfurt am Main, Germany; 3 Calleva Centre, Magdalen College, Oxford, UK; 4 CAB International, Ascot, UK

**Keywords:** Fungi, Plant pathogens, Fungicide resistance, Effector genes, Culture collections, Evolution, Genomics

## Abstract

**Background:**

Fungal pathogens are major contributors to global losses of crop yields. Despite large-scale efforts to develop fungicides and resistant plant genotypes, disease outbreaks still pose severe risks to food security due to fungicide resistance and high adaptability of fungal pathogens. Genetic mechanisms behind the acquisition of resistance and renewed virulence have been uncovered by genome sequencing, especially of pathogens of main crops targeted by major control programs. Here, we investigate the use of comparative genomics of historical isolates to investigate how the wider community of fungal plant pathogens evolved during agricultural intensification.

**Results:**

We analysed historical cryopreserved fungal isolates from three species that were collected in the UK between 1950 and 2000. Comparative genomics of 32 genomes was used to identify variable genome regions that represent putative targets of strong selection during this period, focusing especially on targets of fungicides and putative effector genes that might underpin changes in virulence. Using methods suitable for isolate rather than population sampling, we found evidence of rapid changes in single nucleotide polymorphism frequency in a suite of genes involved in pathogenesis, which overlapped partly between two of the species. We also found turnover in effector gene content in the UK during the period, but generally conserved evolution of fungicide target genes. Sample time and host explained similar amounts of variation in both single nucleotide polymorphism (SNP) changes and variation in effector gene content.

**Conclusions:**

The described approach could be scaled up in the future to reconstruct the evolution of hundreds of species and samples held in historical fungal collections worldwide throughout the course of the Green Revolution.

**Supplementary Information:**

The online version contains supplementary material available at 10.1186/s12864-025-12472-2.

## Background

Fungal pathogens are major contributors to the estimated loss of 20–40% of crop yields, and $220 billion of revenue, to plant diseases annually (FAO, 2022). Since the 1950s, these organisms have faced intense selection pressures from new control efforts such as the development of successive classes of fungicides and selective breeding of resistant crop plants [1, 2]. For example, the percentage of the UK wheat crop sprayed with fungicide rose from 0 to 100% between 1970 and 1980 [3]. Similarly, selective breeding led to an estimated > 50% increase in average yield per hectare in wheat and barley between 1950 and 2000, and > 20% increases for sugar beet, maize and oilseed rape between 1980 and 2000 [1]. Yet, fungal pathogens still cause major losses today, due to their capacity to evolve resistance to new chemicals and renewed virulence against previously resistant plant genotypes. Resistance to the single-site fungicides developed from the 1970 s onwards typically involves the spread of single nucleotide non-synonymous resistance mutations in target genes within 10 years [4–6]. In contrast, changes in virulence often involve new combinations of effector genes, associated with rapidly evolving genome regions, hybridization and horizontal gene transfer [7–10]. Documenting how these organisms evolved in response to past control measures can provide vital insights into the development of new control strategies.

Historical isolates in culture collections provide a useful resource for tracking evolution during the past [11–13]. By spanning the critical period that saw the spread of fungicide use and turnover in crop varieties, these isolates can reveal which genome regions responded to intense selection during that period. Contrary to collections of preserved plants and animals, historical fungal isolates can be revived from cryopreserved spores, thus offering fresh tissue for high-quality DNA extraction and genome sequencing [13]. For example [11, 12], sequenced isolates of Coffee Wilt Disease, *Fusarium xylarioides*, collected over a span of 52 years before and after known outbreaks to show how horizontal gene transfer led to changes in effector gene composition between outbreaks. Culture collections do have some limitations for population analyses, because they comprise mostly single isolates sampled haphazardly with respect to location, time and host, rather than a random or structured population sample. Nonetheless, the time dimension and breadth of sampling across taxa offer the potential for insights that complement detailed studies of populations [14] and fitness consequences of resistance mutations [2, 15, 16].

Here, we investigate the potential for comparative historical genomics of plant pathogenic fungi using cryopreserved strains from culture collections. We focus primarily on the United Kingdom as a sampling region and selected three generalist taxa with sample dates ranging from the 1950 s to the late 1990 s in the IMI Genetic Resources Collection of CAB International (CABI). *Fusarium culmorum* (Wm.G. Sm.) Sacc. causes *Fusarium* Head Blight (FHB) and crown rot in many cereals [17–19]. As a target of direct control, it likely was exposed to successive fungicides as well as rapid turnover of genotypes on wheat and rye during this interval [20–22]. Previous studies have investigated its resistance to demethylation inhibitor (DMI) fungicide [23], genomics and metabolomics [24]. *Verticillium nonalfalfae* Reinke & Berthold causes wilt in potatoes [25, 26], hops [27, 28], and more recently in *Ailanthus*, which are invasive trees in the USA [29–31]. As a soil-borne pathogen, this species has likely experienced less intensive control measures than those found on cereals. Finally, *Fusarium lateritium* Nees infects many tree species [32], causing fruit rot in e.g. plums and peaches [33, 34], canker disease, shoot dieback in *Dalbergia* and *Acer* [35, 36], and even infects humans in isolated cases [37, 38].

We ask whether we can detect responses to agricultural intensification over this period by searching for targets of selection across the whole genome, focusing on known fungicide target genes and turnover of putative effector genes. Comparison of three species allows the search for overlaps in cases of rapid change between species, which might arise due to shared selection pressures. If our assumptions about the intensity of control on relevant hosts are correct, however, we might expect more evidence of strong selection in *F. culmorum* than *V. nonalfalfae* than *F. lateritium*.

## Methods

### Sample selection

A total of 32 fungal isolates from three species, *Fusarium culmorum*, *Verticillium nonalfalfae* and *Fusarium lateritium*, were obtained from the CABI-IMI Genetic Resources Collection (Table 1). These species and isolates were chosen based on the availability of isolates across different decades between the 1950 s and 2000, focusing mainly on the UK to minimize potential effects of geographical population differentiation. Plant hosts varied and were recorded in the CABI-IMI metadata (Table 1).

**Table 1:** Sample information including genome assembly statistics. More details in Supp. Table 1

Species	IMI accession	Collection year	Host	Accession no.	Assembly size (Mb)	No. of contigs	N50 (kb)	GC content (%)	Complete BUSCO %
*Fusarium lateritium*	126338	1967	*Corylus*	JBIPBZ000000000	39.9	270	449	48.11	99.66
	172217	1972/ 1973	*Sarothamnus scoparius*	JBIQGC000000000	37.7	368	403	47.44	100
	250545	1980	*Clematis*	JBIPCA000000000	37.9	1444	75	48.72	100
	300533	1982–1990	N/A	JBIQGD000000000	37.6	99	2985	47.6	100
	301104	1986	*Malus pumila*	JBIQGE000000000	40.7	356	568	47.44	99.66
	317237	1987	*Fraxinus*	JBIQGF000000000	39.5	284	448	48.19	100
	332722	1989	*Salix*	JBIPCB000000000	37.7	611	156	48.63	99.66
	353158	1992	*Robinia pseudoacacia*	JBIPCE000000000	39.8	386	462	48.17	99.66
	356570	1992/ 1993	*Fraxinus excelsior*	JBIQGG000000000	38.3	296	412	48.29	100
	362216	1994	*Ruscus aculeatus*	JBIPCF000000000	37.8	215	665	47.55	100
	380165*	1997	*Malus domestica*	JBIPCG000000000	39.8	460	307	48.24	99.66
*Fusarium culmorum*	113133	1957	*Poaceae*	JBIQFV000000000	37.8	587	1446	47.76	99.66
	135794	1968	*Zea mays*	JBIQFW000000000	37.5	738	1551	47.78	99.66
	159025	1971	*Triticum*	JBIQFX000000000	39.9	1372	598	47.92	99.66
	202042	1976	*Hordeum*	JBIQFY000000000	36.6	448	1580	48.12	99.66
	270555	1982	*Triticum*	JBIQFZ000000000	36.7	580	1968	47.97	99.66
	309752	1986	*Triticum*	JBIQGA000000000	36.9	457	1563	48.1	99.66
	336336*	1989	*Euphorbia pulcherrima*	JBIQGB000000000	38.7	612	1144	48.05	99.66
*Verticilium* *nonalfalfae*	62131	1956	*Lycopersicon esculentum*	JBIQGN000000000	32.6	659	472	55.64	99.31
	62464	1956	N/A	JBIQGO000000000	33.1	675	353	55.37	99.31
	172737	1971/ 1973	*Lycopersicon esculentum*	JBIQGQ000000000	33.0	704	330	55.65	98.97
	172738	1971/ 1973	*Lycopersicon esculentum*	JBIQGR000000000	32.8	675	295	55.89	99.31
	172739	1971/ 1973	*Lycopersicon esculentum*	JBIQGS000000000	32.9	687	370	55.83	99.31
	172746	1971/ 1973	*Lycopersicon esculentum*	JBIQGT000000000	33.1	754	446	55.56	99.31
	295224	1985	N/A	JBIQGU000000000	33.7	121	491	55.35	98.97
	298092	1985	*Humulus lupulus*	JBIQGV000000000	32.6	636	390	55.9	99.31
	298093	1985	*Humulus lupulus*	JBIQGW000000000	32.7	620	317	55.78	99.31
	298097	1985	*Humulus lupulus*	JBIQGX000000000	32.8	666	308	55.75	99.31
	298098	1985	*Humulus lupulus*	JBIQGY000000000	33.3	797	347	55.71	99.31
	298102	1985	*Humulus lupulus*	JBIQGZ000000000	33.6	1196	477	55.5	99.31
*Verticillium albo-atrum*	278734	1983	*Solanum tuberosum*	JBIPCI000000000	35.9	808	112	54.47	99.66
	331060*	1989	*Fragaria*	JBIPCJ000000000	35.6	88	1482	56.46	95.86

*used as reference genome for read mapping and SNP calling. *V. albo-atrum* reference used for *V. nonalfalfae*

### Cultivation and DNA extraction

Cryopreserved fungal spores were rehydrated with distilled water for 30 min and spread on potato dextrose agar plates. Once the isolates established mycelial growth, they were checked for contamination under a light microscope. Uncontaminated plates were sub-cultured onto cellophane-lined potato dextrose agar plates. All agar plates were kept at 21 °C (room temperature). Mycelia were harvested, snap-frozen in liquid nitrogen and ground with 2 mm steel beads using the TissueLyser II (QIAGEN, Germany). DNA was then extracted using the DNeasy Plant Mini Kit (QIAGEN, Germany) following the manufacturer’s protocol, except with 30 min of incubation with the lysis buffer at the start. Purified extracted DNA was sent to Oxford Genomics Centre (Oxford, UK) for sequencing library preparation and 150 bp pair-ended sequencing on the Illumina NovaSeq 6000 platform.

### Raw data filtering and genome assembly

Adapters were removed from raw reads using cutadapt v3.7 [39], followed by low-quality reads using trimmomatic v0.39 [40] with default settings. FLASH 1.2.11 [41] was used to create longer contigs, with both extended and non-extended fragments then used for genome assembly with SPAdes v3.13.0 [42] using default parameters. Blobtools v1.1 [43, 44] was used to identify contaminated scaffolds in the genome assemblies against the NCBI nt database. Scaffolds that were not assigned to the respective genera of the isolates (*Fusarium*, *Verticillium*) were removed (these were exclusively bacterial contaminants of grown cultures). Genome assembly quality and completeness were assessed with QUAST v5.0.2 [45] and using the fungi_odb10 database in BUSCO; [46] before and after filtering of contaminated scaffolds.

### Comparative genomics

Annotation was conducted with funannotate v1.8.10 pipeline [47] to compare genomes of the same species. First, the *funannotate clean* script was used to remove duplications. Then, the *funannotate sort* script was used to sort the scaffolds by size and rename them to NCBI-compatible standards, followed by *funannotate mask* to mask repeat contents in the genomes. The *funannotate predict* script was used for whole-genome gene predictions using various incorporated tools, including Augustus v3.3.3 [48] and GeneMark-ES v4.69_lic [49].

Additional functional annotations of the genomes were carried out using the online versions of phobius [50], antiSMASH 6.0 Fungi [51], as well as InterProScan5 v5.60–92.0.0 [52] through the *funannotate remote* script. The *funannotate annotate* script was then used to add all annotations to the prediction results from the previous step. Finally, the *funannotate compare* script was used to compare the annotations from all isolates within the same species (including Pfam, Interpro, CAZy and MEROPS) for the analysis of protein or enzyme family expansion, contraction and presence/absence, and non-metric multidimensional scaling (NDMS) for Pfam domains and Interpro families. As crop pathogens are known to have extremely specialized genomes due to strong local adaptation (that leads to gene duplication or deletion [53–56] and chromosomal rearrangements [57, 58], we used our de novo assemblies rather than mapping reads onto public reference genomes to retain as many genomic sequences as possible. OrthoFinder v2.5.4 [59] was used to assign genes to orthologous groups within the same species and reconstruct phylogenetic trees (more details below).

### Tests for genetic structure and sub-populations

To test for population structure and cryptic species we used the consensus ‘species tree’ inferred from all orthologous genes using the STAG algorithm [59] and rooted using the STRIDE algorithm [60] in Orthofinder to test for major subdivisions within each of our three focal taxa. If all the isolates within a taxon belong to the same species, and with sufficient rates of sex and recombination, then ancestry should vary across unlinked loci and yield discordance and poor resolution in the consensus ‘species tree’. We therefore tested for genetic structure and presence of sub-populations by searching for nodes that are supported by a high proportion of gene trees (> 60%), indicative of genealogical concordance and the separation of two isolated populations or cryptic species [61]. We also used clustering of isolates based on protein presence/absence metrics to identify any substantial structure in gene presence or absence, which could also indicate divergent populations or cryptic diversity within the species – especially if associated with differences in host plant between divergent clusters.

### Searching for SNPs displaying rapid mid-time period changes

Reads were mapped back onto a single genome chosen as a ‘reference’ for each species (Table 1) using Burrows-Wheeler Alignment (BWA) software, and single nucleotide polymorphisms (SNPs) were called using bcftools 1.9 [62] with default settings. The sequenced genomes should all be haploid and we filtered out SNPs with intermediate frequencies within a single sample using the vcfR library in R. SNPs with mapping quality scores < 35, a call of “NA” in any taxon, a call of allele 1 when mapping the reference taxon reads back onto its own genome were also filtered to focus on robustly called variants.

SNPs with at least 3 counts of the minor allele were used for further analysis, to have sufficient power, at least in principle, to detect a large change in frequency over time. Our sample lacks the power to detect small changes in allele frequency, but we could detect strongly selected changes with a rapid change in frequency; for example, where the first *n* samples display biallelic variant 0 and the last *m* samples display variant 1, as might be expected with the rapid spread to fixation of a resistance mutation during the rise of chemical fungicide applications in the 1970s. We fitted binomial generalized linear models of SNP frequency over time to rank SNPs according to evidence for a rapid change in the mid-time period for each species. For each model, the response variable was the allele of each isolate, taking values of 0 or 1, and the explanatory variable was sampling year of the isolate, assigning binomial errors using the family option in the glm function in R. We compared the frequency of large changes across all SNPs to the proportion expected under randomization of isolates among time points, i.e. under the null model that SNP frequency is independent of time. The putative function of genes containing large frequency changes in coding SNP variants was recorded.

Because changes in fungal virulence are often associated with changes in gene content in dynamic genome regions, we repeated the analysis for indel variants (of 2 or more base pairs in length), running binomial regressions to detect indel variants showing large changes in frequency over time.

### Variability in fungicide resistance genes

We extracted sequences of genes that are known to be fungicide targets from all of our genomes using BLAST + 2.13.0 [63]: cytochrome b, targeted by quinone outside inhibitors (QoI, introduced in the 1990 s), beta-tubulin targeted by methyl benzimidazole carbamate fungicides (mbc, introduced early 1970 s), and sterol 14-demethylase (CYP51 genes) targeted by azoles (introduced during the 1970 s). The Fungicide Resistance Action Committee (FRAC) database lists cases of resistance in *F. culmorum* to mbc fungicides but does not report cases of resistance to any fungicide in *V. nonalfalfae* (although a case in *V. albo-atrum* is reported) or *F. lateritium*. We recorded any polymorphism in sites previously associated with fungicide resistance mutations in the literature. If our taxa evolved resistance over this time, we predicted that samples prior to the first use of each fungicide class should lack resistance mutations, whereas later samples could contain them.

We aligned gene copies using MAFFT v7.505 (Katoh and Standley, 2013). For any with multiple polymorphic sites present, we reconstructed gene trees using IQTREE2 choosing the best model with ModelFinder [64] and tested for positive selection on amino acid sequence using PAML v4.10.6 [65]. We compared a model with conserved and neutral categories of codons (dN/dS ratio < 1 and = 1 in turn, model 1 A) to a model with an extra category of sites under positive selection (model 2 A). Log likelihood ratio tests were used to test for the significance of model 2 A over model 1 A. We repeated tests on a broader set of genes belonging to clusters of orthologous genes (COG) category Z, cytoskeleton, which beta-tubulin belongs to, and COG category Q, secondary metabolites, which CYP51 belongs to, to contextualise the analyses of the target genes.

### Variation in effector gene content

We used SignalP v6.0 [66] to identify the secreted proteins and ran EffectorP 3.0 [67] to identify putative effectors from our proteomes. We quantified the presence and absence of those genes across samples within species to identify putative effectors that were apparently gained or lost during the time interval. To refine inferences on gain and loss, we double-checked for the presence and absence of putative effectors based on orthogroup clusters using BLAST [63] (pipeline shown in Fig. S1). Finally, we tested whether the rate of gain and loss of effectors was higher than for core genes that are not putative effectors using randomization tests.

## Results

### Genome quality and gene content

We sequenced seven isolates of *Fusarium culmorum* (1957–1989), 12 isolates of *Verticillium nonalfalfae *[83] and 11 isolates of *Fusarium lateritium* (1967–1997). All assembled genomes have more than 99% complete BUSCO genes except for three *V. nonalfalfae* isolates (all > 95%, Table 1). Although the number of contigs and N50 contig length varies within each species, assembled genomes size and GC content is consistent, ranging from 36.6 to 37.8 Mb and 47.76–48.12% for *F. culmorum*, 32.6–35.9 Mb and 54.47–56.46% for *V. nonalfalfae*, and 37.6–40.7 Mb and 47.4–48.7% for *F. lateritium* (Table 1). Numbers of genes, proteins and tRNAs were largely consistent within species, although *V. nonalfalfae* and *F. lateritium* contained 2 and 4 isolates, respectively, with a higher number of unique proteins than the rest (Fig. S2, Table S1), which corresponds to deep phylogenetic branching.

### Evidence of population structure or cryptic species within taxa

**Fig. 1:**
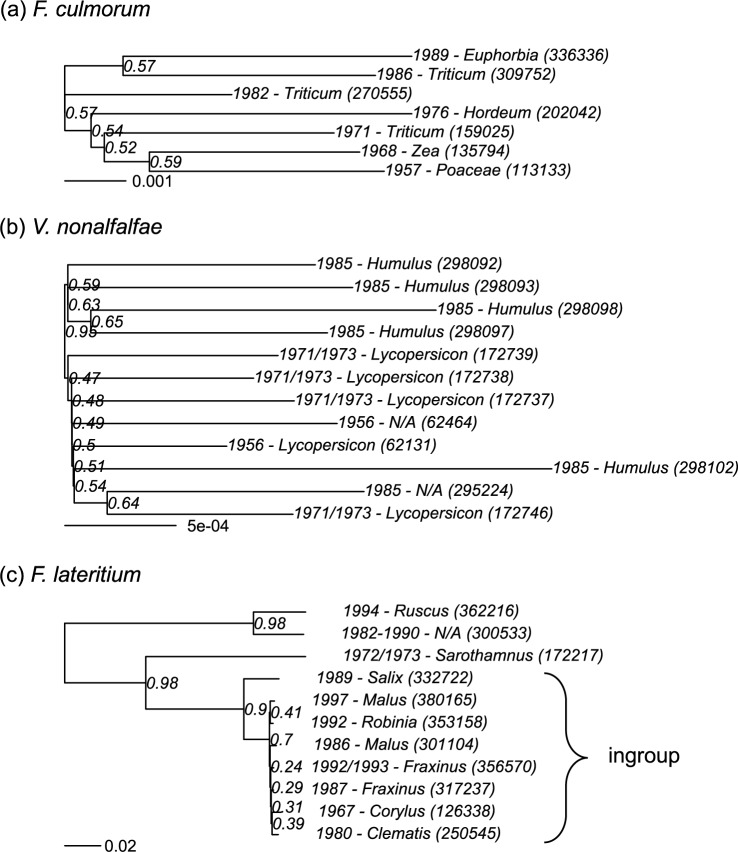
Species trees for (**a**) *F. culmorum*, (**b**) *V. nonalfalfae* and (**c**) *F. laterititium* based on 9356, 6778 and 7389 single-copy orthologous gene trees respectively. Reconstructions used the STAG algorithm and were rooted using the STRIDE algorithm (Emms and Kelly, 2017), both implemented in OrthoFinder [59]. Support values representing the proportion of gene trees supporting the depicted partition are indicated at each node. Branch lengths are in number of substitutions per site, averaged across all individual gene trees. All samples are named by year of collection, host plant and IMI accession number in brackets

**Fig. 2:**
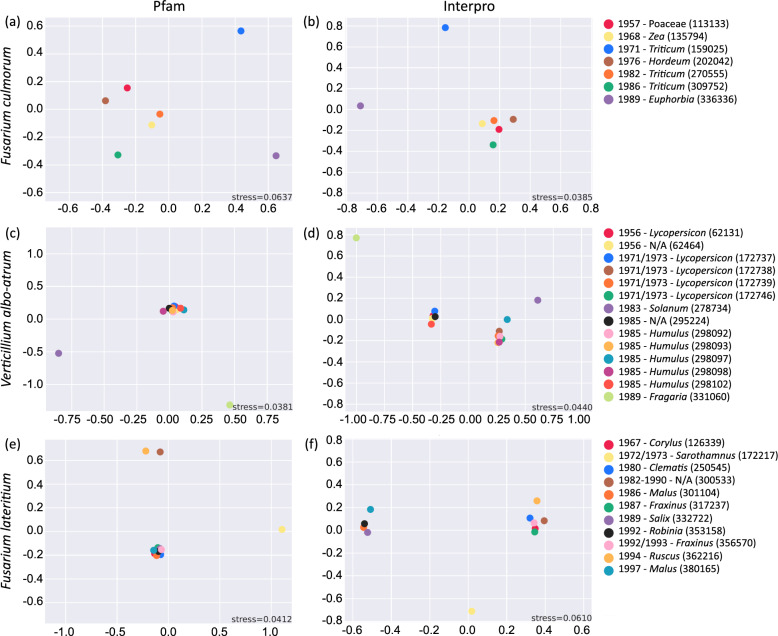
NDMS plots for Pfam and Interpro families for each species. **a**) and **b**): *F. culmorum*, **c**) and **d**): *V. nonalfalfae*, **e**) and **f**): *F. lateritium*. Plots were generated with the *funannotate compare* script. All samples in the key are labelled by year of collection, host and IMI accession number in brackets

*Fusarium culmorum* isolates display low genetic variation and a lack of deep phylogenetic structure in the ‘species tree’ reconstructed from gene trees of single-copy orthologs (Fig. 1A). No internal nodes are supported by > 60% of gene trees. Non-metric multidimensional scaling (NMDS) of Pfam domains and Interpro families similarly shows the isolates evenly distributed without evident sub-clusters (Fig. 2A, B). In *V. nonalfalfae*, isolates comprise a little-differentiated clade with either *L. esculentum* or *H. lupulus* as hosts (plus one isolate with no host record, Fig. 1B). Two isolates identified as *V. albo-atrum* sensu stricto were phylogenetically divergent from the *V. nonalfalfae* isolates and excluded from further analyses. In *F. lateritium*, three isolates either on shrubs or with no host record are phylogenetically divergent from an undifferentiated clade sampled from trees and a clematis (Fig. 1C, > 10% pairwise nucleotide divergence across orthologous genes and > 90% of gene trees across orthologs support the same grouping). In both *V. nonalfalfae* and *F. lateritium*, NMDS of Pfam domains supports similar clustering to the species tree for orthologs, whereas NMDS of Interpro families display alternative or additional sub-clustering, but in neither case simply separated by host plant (Fig. 2C-F).

### Rapidly changing SNP and indel variants

**Fig. 3:**
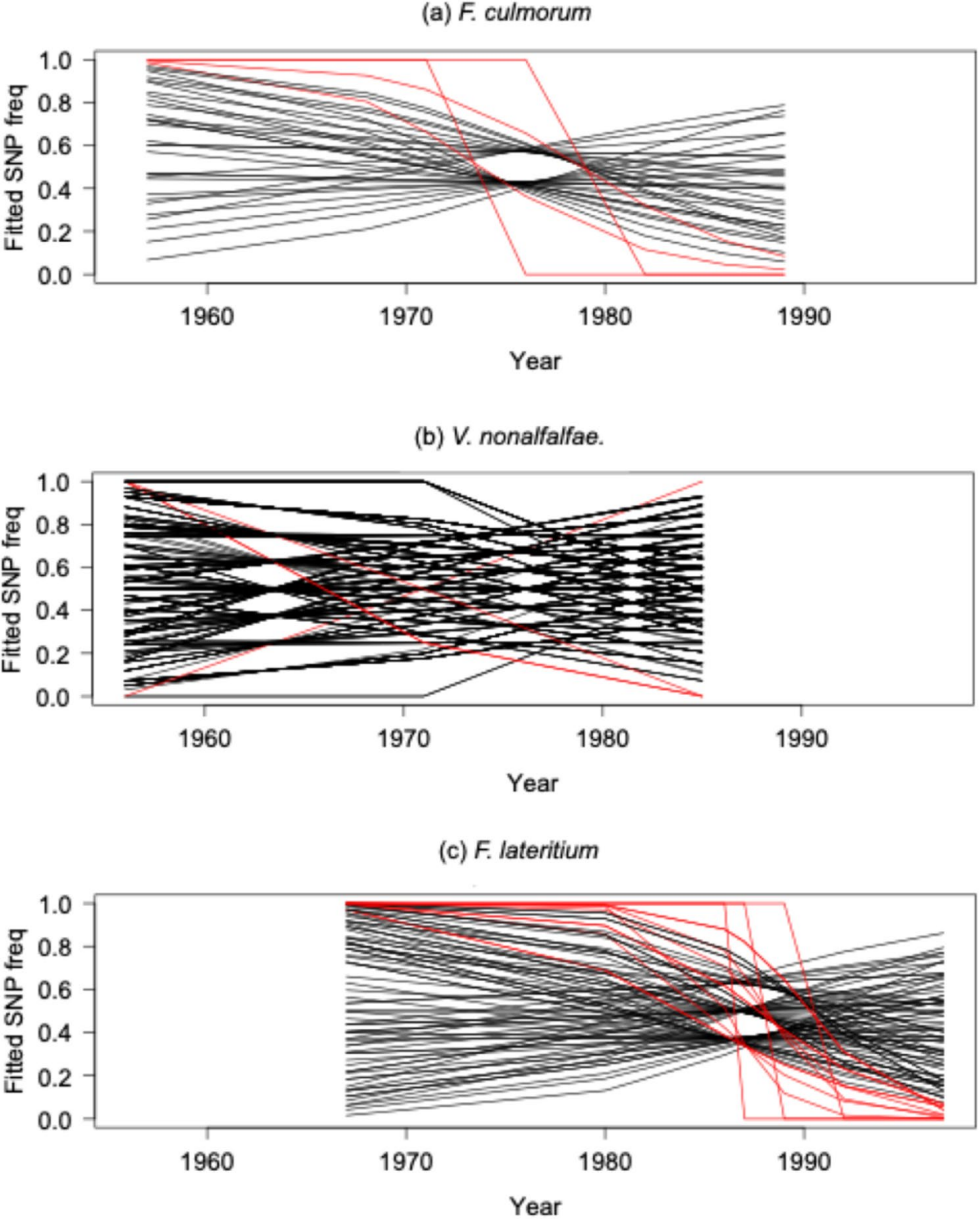
Predicted changes in frequency for all Single Nucleotide Polymorphisms (SNPs) to identify rapidly changing SNPs in each species. SNPs were filtered as described in the materials and methods, then binomial linear models were used to predict the changes in frequency over time. Lines show the predicted frequency of allele 1 versus allele 0 (where 0 is the allele of the designated reference genome for each species). Red lines highlight SNPs with > 0.9 change in allele frequency during the time window sampled for each species. Black lines indicate SNPs with < 0.9 change in allele frequency: inflection points such as around 1965 for *V. nonalfalfae* occur at the midpoint between available sampling times. Note that each line represents multiple overlapping SNPs showing the same pattern of predicted allele frequencies

We tested for SNPs changing rapidly in frequency over time just among the isolates belonging to the undifferentiated ingroup in each species to avoid confounding effects of population structure or cryptic species: all 7 isolates for *F. culmorum*, 12 for *V. nonalfalfae*, and 8 for F. *lateritium*. The mid-point of available sampling occurs in the 1970 s for the first two species, but in the 1980 s for the last species (Fig. 3). In *F. culmorum*, 2423 out of 28,717 analysed SNPs (8.4%) displayed a predicted change of frequency over time of 0.9 or more (red lines, Fig. 3A, SNP patterns in Fig. S3, Table S2). This proportion is not significantly greater than expected from a null distribution obtained by randomizing sampling time across isolates (*p* = 0.43 of null trials display 8.4% or more of SNPs with a predicted > 0.9 change in frequency, Fig. S4A). Fewer rapidly changing SNPs were identified in *V. nonalfalfae* (Fig. 3B; 4 out of 569 analysed SNPs, 0.7%, had a predicted change in frequency of 0.9 or more, *p* = 0.63 compared to null trials randomizing sampling date among isolates, Fig. S4B), whereas more were detected in *F. lateritium* (Fig. 3C; 26049 out of 189970 analysed SNPs, 13.7%, had a predicted change in frequency of 0.9 or more, *p* = 0.64 compared to null trials randomizing sampling date among isolates, Fig. S4C). Consequently, while we can detect SNPs that seemed to display a large change of frequency over time, the number of such SNPs was not significantly greater than expected if genotypes were randomly distributed across time points; and hence could reflect the expected number of false positives for each separate analysis. Similar patterns were observed for indel variants (*F. culmorum* 89/874, 10.2%; *V. nonalfalfae* 4/273, 1.5%; and *F. lateritium* 1499/9905, 15.1% of variants predicted to change by > 0.9, but proportion not significantly greater than null model, all *p* > 0.5, Fig. S5).

**Fig. 4:**
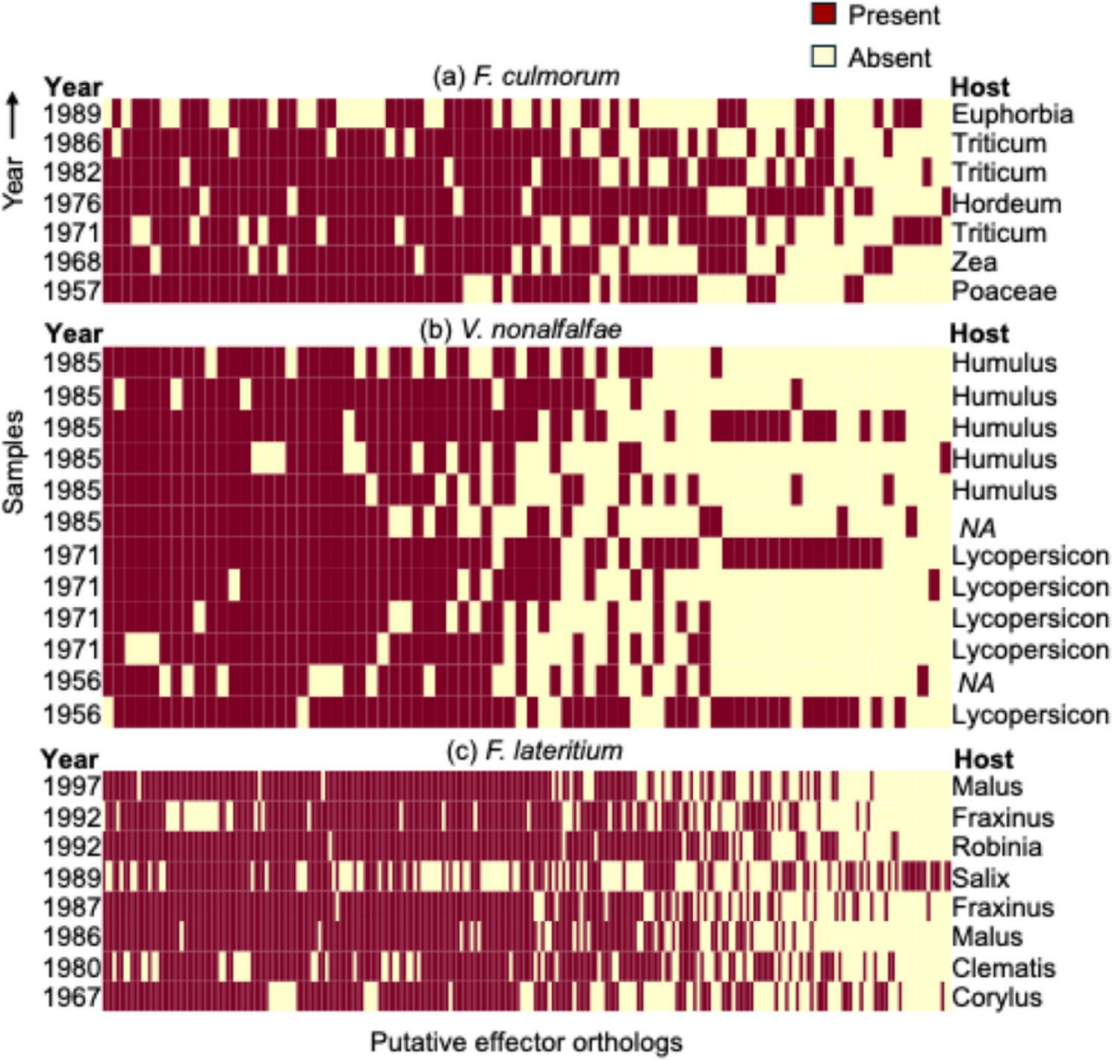
The presence and absence of putative effectors in each species. Only putative effectors with variable presence and absence among samples are shown. In each plot, each row is a fungal isolate, sorted by sampling year (top row = most recent sample). The host plant genus is shown to the right of the plot

We examined the characteristics of the SNPs showing > 0.9 change in frequency to see if there were any shared patterns among the species (Table S3, S4). Of SNPs showing > 0.9 frequency change, 51% occurred outside genes, and among those within genes, 41.3%, 22.4% and 36.4% were 1 st, 2nd and 3rd codon position sites (Table S3). Ten gene ontology (GO) categories enriched among SNPs changing by > 0.9 frequency overlapped between *F. culmorum* and *F. lateritium* (table S4, there were too few SNPs in *V. nonalfalfae* to perform enrichment analysis), which increase confidence that these are biologically relevant changes and not simply false positives due to the low number of samples. These included oxidoreductase activity acting on CH-OH groups (i.e. alcohol oxidases), iron ion binding, flavin adenine dinucleotide (FAD)-binding, carbohydrate binding, fatty acid biosynthesis, glutathione metabolism, and extracellular and membrane cellular components (Table 2). Additional categories were specific to either species (Table S4): *F. culmorum* SNPs were enriched for categories including autophagy, catechol metabolism, beta-galactosidase and cysteine-type endopeptidase activity, and *F. lateritium* for categories for secondary metabolite biosynthesis, ion transmembrane and carbohydrate transporters. The limited annotation information for the four *V. nonalfalfae* rapidly changing SNPs included positive regulation of *Arp2/3* complex-mediated actin nucleation and a gene flagged as a secreted protein.

**Table 2:** Gene ontology categories that were significantly enriched in rapidly changing SNPs in both *Fusarium* species

Shared enriched categories			F. culmorum				F. lateritium				Ref^
number	description	aspect	Count		Frequency	Fisher	Count		Frequency	Fisher	
			Genome	SNPs	Change*	log10p^†^	Genome	SNPs	change	log10p	
GO:0016614	oxidoreductase activity, acting on CH-OH	MF	23	42	4.95E-03	−37.35	27	39	2.52E-04	−3.49	[70, 71]
GO:0005506	iron ion binding	MF	149	30	2.84E-03	−8.04	153	262	1.84E-03	−30.66	[72]
GO:0071949	FAD binding	MF	95	14	1.19E-03	−2.10	99	145	9.44E-04	−13.68	[76]
GO:0030246	carbohydrate binding	MF	32	19	2.13E-03	−10.99	32	38	2.17E-04	−2.45	[73]
GO:0006633	fatty acid biosynthetic process	BP	31	14	1.53E-03	−6.76	39	52	3.21E-04	−4.17	[74]
GO:0006749	glutathione metabolic process	BP	17	12	1.36E-03	−7.24	21	34	2.33E-04	−3.65	[75]
GO:0015205	nucleobase transmembrane transporter	MF	7	4	4.46E-04	−1.57	10	30	2.49E-04	−5.55	[77]
GO:0005576	extracellular region	CC	99	15	1.29E-03	−2.42	93	82	3.45E-04	−2.56	[80]
GO:0009100	glycoprotein metabolic process	BP	79	14	1.27E-03	−2.83	80	69	2.81E-04	−2.01	[81]
GO:0031226	plasma membrane	CC	155	21	1.72E-03	−3.06	150	110	3.34E-04	−1.64	[82]

* frequency of GO term among SNPs – frequency across annotated genome

^†^ log10(false discovery rate corrected *p*-value) ^ Reference for potential role in pathogenesis or fungicide resistance

### Low variability in fungicide resistance genes

We find no evidence for positive selection and little evidence of potential resistance mutations in known resistance genes in any of the species. Instead, fungicide target genes show a pattern of purifying selection typical of representative genes from related functional classes. All three species have one copy of cytochrome b (a mitochondrial gene) targeted by QoI (quinone outside inhibitors) fungicides. *Fusarium culmorum* and *F. lateritium* both have two copies of beta-tubulin targeted by mbc (Methyl Benzimidazole Carbamates) fungicides, and three copies of the CYP51 (CYP51A, B and C, cytochrome P450, Family 51) gene targeted by azoles, whereas *V. nonalfalfae* has one copy of beta-tubulin and one copy of CYP51. Across six sites in cytochrome b with reported resistance mutations against QoI fungicides [68, 69], only wildtype amino acids were present across all samples of all three species. In beta-tubulin, one polymorphism was observed in *V. albo-atrum* 331,060 (from *Fragaria* host) in one of four sites reported to convey mbc resistance (mutation E198L, reported in *Fusarium graminearum* previously; Oliver, 2024 #71) but no polymorphism was seen among the *V. nonalfalfae* isolates. The CYP51 sequences were highly divergent from *Aspergillus* taxa used as reference taxa for resistance mutations to azoles (> 15% pairwise amino acid divergence), and so direct matches could not be inferred between variants and resistance mutations.

Analysing variation in the target genes in each species further, *F. culmorum* displayed low genetic variation across all target genes, with zero, zero, zero, zero and one (synonymous) polymorphic sites in cytochrome b, CYP51A, CYP51B, and beta-tubulin 1 and 2, respectively. CYP51C was more variable and had a relatively high dN/dS ratio (w0 = 0.35, p0 = 1, model 1a) but low absolute numbers of both nonsynonymous and synonymous polymorphisms (mean pairwise amino acid divergence = 0.4%, no evidence for positive selection, log likelihood ratio test, *p* = 1.00, Table 3). A similar pattern was observed across genes in the COG Q and Z pathways, the average dN/dS was relatively high (mean w0 = 0.35 +- 0.056 S.E., Fig. S6, Table S5), but pairwise amino acid divergence low (1.8+- 1.1% *n* = 36 genes) with little evidence for positive selection (log likelihood ratio test, all *p* > 0.05). *V. nonalfalfae* had one copy of erg11 and one copy of beta-tubulin. There was a dominant pattern for strong purifying selection on all target genes (mean w0 = 0.0097+−0.0033 across 3 target genes) and on the COG sets of genes (mean w0 = 0.043+- 0.014, *n* = 12 genes, Fig. S6). *F. lateritium* has three erg11 copies and two beta-tubulin copies, with a pattern of purifying selection on all of them (mean w0 = 0.029+−0.0039), and on the COG sets of genes (mean pairwise AA = 16.7+−1.7%, mean w0 = 0.045+−0.0032, *n* = 41 genes, Fig. S6, table S5).

**Table 3:** Genetic variation and codon substitution models for fungicide target genes

Species	Gene	Model	Proportion of codons			dN/dS ratios			Log Likelihood	*p*	Pairwise divergence/site	
			p0	p1	p2	w0	w1	w2			Amino acid	Nucleotides
*F.culmorum*	CYP51C	1a	0.99999	1e-05		0.34511	1		−2063.75	1.00	0.0040	0.0031
*F.culmorum*	CYP51C	2a	1	0	0	0.34512	1	1.00	−2063.75			
*V. nonalfalfae*	cob	1a	0.9914	0.0086		0.01834	1		−1745.21	1.00	0.0191	0.0446
*V. nonalfalfae*	cob	2a	0.9914	0.00056	0.00803	0.01834	1	1.00	−1745.21			
*V. nonalfalfae*	CYP51	1a	0.96305	0.03695		0.01047	1		−2812.20	1.00	0.0660	0.1259
*V. nonalfalfae*	CYP51	2a	0.96305	0.01264	0.02431	0.01047	1	1.00	−2812.20			
*V. nonalfalfae*	TUB	1a	0.98654	0.01346		0	1		−1815.19	0.12	0.0087	0.0605
*V. nonalfalfae*	TUB	2a	0.99034	0	0.00966	0.00055	1	5.22	−1813.08			
*F. lateritium*	cob	1a	0.98378	0.01622		0.01959	1		−1801.96	0.36	0.0104	0.0273
*F. lateritium*	cob	2a	0.99684	0	0.00316	0.02649	1	9.22	−1800.93			
*F. lateritium*	CYP51A	1a	0.97072	0.02928		0.03664	1		−2315.02	1.00	0.0543	0.0872
*F. lateritium*	CYP51A	2a	0.97072	0.00911	0.02017	0.03664	1	1.00	−2315.02			
*F. lateritium*	CYP51B	1a	0.97063	0.02937		0.04421	1		−3445.35	1.00	0.1025	0.1670
*F. lateritium*	CYP51B	2a	0.97063	0.01415	0.01522	0.04421	1	1.00	−3445.35			
*F. lateritium*	CYP51C	1a	0.96473	0.03527		0.02981	1		−4229.47	1.00	0.0591	0.1273
*F. lateritium*	CYP51C	2a	0.96473	0.01909	0.01618	0.02981	1	1.00	−4229.47			
*F. lateritium*	TUB1	1a	0.9805	0.0195		0	1		−1703.75	0.80	0.0008	0.0097
*F. lateritium*	TUB1	2a	0.99687	0	0.00313	0	1	18.63	−1703.52			
*F. lateritium*	TUB2	1a	0.99999	1e-05		0.01041	1		−597.89	1.00	0.0106	0.0560
*F. lateritium*	TUB2	2a	1	0	0	0.01041	1	1.00	−597.89			

### Turnover of putative effector genes

SignalP v6.0 identified on average 1082, 970 and 1057 secreted proteins per genome of *F. culmorum*, *V. nonalfalfae* and *F. lateritium*, respectively. From these, a combined total per species of 1025, 1187 and 1454 genes were recognised as effectors by EffectorP v 3.0. Refining presence or absence in the original Orthofinder assignments using a BLAST pipeline resulted in, on average, 3, 2 and 1 genes being added back per sample in each species (i.e. putatively missing due to annotation or orthogroup assignment errors, but discoverable in the genome of that isolate using blast).

In *F. culmorum*, 938 putative effectors (91%) were shared by all samples (Table S6). Most of the core effectors (587, 63%) were under strong purifying selection with dN/dS ratios less than 1, while 160 of them had identical sequences, rendering the dN/dS ratio undefined. The remaining effectors showed evidence of positive selection (dN/dS > 1, 195, 20%). Apart from the large fraction whose functions remained unknown, hydrolases appeared to be a dominant category among core effectors with putative functional annotation. A remaining 87 putative effectors were variably present or absent among samples (8% of all effectors, Fig. 4). Many of the putative effectors belong to the broad category of carbohydrate active enzymes (CAZymes), such as cellulase and glycoside hydrolase. In contrast, in both *V. nonalfalfae* and *F. lateritium*, a lower fraction of putative effectors was shared among all samples (75% and 57% respectively), but those core effectors show an overwhelming pattern for purifying selection (> 98% of core effector genes in both species), compared to *F. culmorum*. *V. nonalfalfae* shows a similarly low proportion of putative effector orthologs with variable presence/absence (6%), whereas the proportion is higher in *F. lateritium* (17%).

On average, 15.5%, 10.2% and 14.1% of the variability in putative effector presence or absence in *F. culmorum*, *V. nonalfalfae* and *F. lateritium* was explainable by sampling year (generalised linear models, binomial error structures, variation summarised as % total deviance explained, averaged across all effector orthogroups), compared to 12.3%, 12.2% and 14.1% explained by host. Some isolates on divergent hosts seemed to have a divergent effector gene profile; for instance, the *F. culmorum* strain IMI 336,336 sample from *Euphorbia* (a dicot) had more gene absences than the other samples from cereals. Similarly, the *F. lateritium* isolated from *Salix* sp. had more gene absences than the others. However, three divergent *V. nonalfalfae* that shared effectors not found in the other isolates were isolated from both hops and tomatoes, the two main host species. In the study here, sampling year and host had similar correlations with effector presence/absence variation, leaving a residual 70% of variation unexplained. Further analysis of isolates from different host plants would be needed to further quantify the role of the host in shaping variability.

Among the orthologs that correlated with year or host (1 with year in *F. culmorum*; 3 with host/year in *V. nonalfalfae*, as host and sampling year covaried strongly; and 5 with year and 5 with host in *F. lateritium*), most were annotated as ‘hypothetical protein’. The only exceptions are leucine aminopeptidase 1 in *V. nonalfalfae* and an oxidoreductase GO term for a putative effector correlated with year in *F. lateritium*. Elsewhere, we have investigated the broader scale structural variation and gain and loss of large genome regions in *F. culmorum* and *V. nonalfalfae* [83].

## Discussion

We used strains from historical fungal culture collections and whole genome sequencing approaches to investigate the temporal evolution of three fungal plant pathogens. Patterns of SNP, indel and putative effector presence and absence all showed evidence of genetic turnover across our samples. Overall, the proportion of variation explained by time was 11.6–15.2% for SNPs, 9.3–16.2% for indels and 12.2–14.1% for putative effector presence and absence, across the three species. Host explained a similar proportion of variation, but we had low statistical power for host analyses due to the relatively low number of samples per host species in these generalist fungi.

The rapidly changing SNPs (with a modelled change in frequency > 0.9 over time in each species) were significantly enriched for several gene ontology (GO) categories of known relevance for colonization and pathogenesis of plant fungal pathogens. All categories except one have existing evidence in the literature for roles in pathogenesis in plant pathogenic fungi (Table 2). These included functions associated with the acquisition of nutrients from host tissues, such as alcohol oxidases [70, 71], iron [72] and carbohydrate binding [73]; virulence and resistance to plant immune responses, such as fatty acid biosynthesis [74], glutathionine metabolism as stress resistance to host oxidative response [75]; and other functions promoting pathogenesis, such as FAD-binding [76], which is a known cofactor for enzymes used for host colonization. SNPs were also enriched for extracellular and plasma membrane components, which are expected to be the main sites for genes with strong interactions with the host and its defences. The final shared category, nucleobase transmembrane transporter genes, are required for sensitivity to Succinate Dehydrogenase Inhibitor fungicides (SDHIs) in *Aspergillus nidulans* [77]. Although further work would be needed to confirm the functional significance of the observed changes, the correspondence to known functions supports our approach as a *de novo* method to identify genes of interest. The wider enriched categories could be investigated in future by large samples of each fungal species (e.g. extending to global scales), testing for replicated trends across a larger sample of species, or with functional genetic confirmations in the laboratory and *in planta*.

Contrary to our hypothesis that evolutionary changes would be faster in *F. culmorum* than *V. nonalfalfae* than *F. lateritium* due to more active pathogen control measures, we found the highest proportion of SNPs, indels and putative effector genes changing over time in *F. lateritium*, and the lowest in *V. nonalfalfae*. This could reflect that *F. lateritium* is more diversified than the other species in terms of hosts, as it was isolated from more host species. *F. culmorum* in the UK displays extremely low genome variation, with little variation across samples spanning 32 years that overlap the development of new classes of fungicides deployed widely in cereal crops, such as mbcs and DMI (azoles). Despite our prior expectation that resistance mutations should arise over time in this population, we found little variation in fungicide resistance target genes, which primarily displayed evidence of strong purifying selection. The lack of evidence for strong selection in targets of fungicide resistance is potentially explained by low intensity of fungicide applications (especially for *F. lateritium* or *V. nonalfalfae* on lower intensity crops) or alternative modes of resistance evolved (such as efflux pumps) rather than target site mutations or the pathogen populations already had natural tolerance to fungicides. For instance, the two *Fusarium* species studied here have duplicated fungicide target genes that could potentially enhance resistance, but copy number was constant among our samples.

Our approach assumes that the genetic content of the samples regrown from the cryopreserved collections still reflects the gene content of the original samples isolated from pathogenic material on plants. Although we did not perform assays to determine if frozen cultures retained the ability to infect host plants here, similar studies elsewhere have managed to infect host plants with material frozen for many decades and fail to infect alternative host plants (Peck et al. 2024). The number of generations involved in original isolation and preparation of material for freezing should be low enough that genetic changes in the laboratory are minimal. Analyses of gene gain and loss further assume that ‘missing’ genes are truly absent and not missing from a particular isolate due to genome assembly or ortholog-assignment errors. BUSCO scores > 99% support high levels of completeness of our genomes for core low-copy genes. Still, searching for ‘missing’ genes in assemblies using BLAST added in between 1 and 3 effector genes per isolate in these species, which demonstrates the importance of checking gene presence/absence patterns.

## Conclusions

So far, relatively few genomics studies have tracked the evolution of single fungal species over time (except that of the bioinsecticide *Beauveria bassiana*; [78]; the causal agent of coffee wilt disease, *Fusarium xylarioides* [12]; see also myxoma virus in rabbits; [79]), and even fewer tracked evolution in multiple species within a similar timeframe. Evolution of fungal pathogens is a complex interplay of spatial heterogeneity, reproductive mode, hybridization, host specificity and many other factors, which ultimately will require large sample sizes to disentangle [83]. Hence, this study serves as a first step in identifying common evolutionary trajectories among different species, as well as exploring the potential of using historical collections in understanding the evolution of fungal pathogens with larger scale studies of more genomes in the future.

## Supplementary Information

Supplementary Material 1.

Supplementary Material 2.

## Data Availability

Genomic data are available in Genbank under BioProject PRJNA1170473, BioSamples SAMN44107511- SAMN44107558 and the short read archive (SRA) accessions SRR30921418- SRR30921471. The BioProject and ranges include additional unpublished genomes, lists for samples used here are in table S1.

## Abbreviations

CABI: CAB international
CYP51: Cytochrome P450, Family 51
DMI: Demethylation inhibitors
FAD: Flavin adenine dinucleotide
FHB: Fusarium head blight
mbc: Methyl benzimidazole carbamates
NMDS: Non–metric multidimensional scaling
QoI: Quinone outside inhibitors
SNP: Single nucleotide polymorphism
