# Supplementary material for: Historical comparative genomics to track the evolution of fungal pathogens: a proof of concept
Source: BMC Genomics. 2026 Jan 12;27:164. doi: 10.1186/s12864-025-12472-2 (PMC12888477; doi:10.1186/s12864-025-12472-2)
Supplement: Supplementary file 1 — Supplementary Material 1. [file 12864_2025_12472_MOESM1_ESM.docx]

**SUPPLEMENTARY FIGURE LEGENDS**

**Figure S1**. Workflow for pipeline to check patterns of presence and absence of putative effectors using BLAST.

**Figure S2**. Number of (a) genes; (b) proteins; (c) tRNAs; (d) unique proteins; and (e) proteins with at least one ortholog in the isolates identified by *funannotate*. Isolates are ordered by IMI accession number (chronologically).

**Figure S3**. The SNP patterns that were associated with a predicted change in allele frequency of 0.9 or more during the sampled time window for each species. SNP patterns were observed across multiple SNPs, especially in *F. culmorum* and *F. lateritium*. Commas are used to show the alleles present for isolates obtained from the same sampling year.

**Figure S4**. The distribution of predicted frequencies of change in biallelic SNPs during the time window sampled for each species. Bars show the observed counts of numbers of SNPs showing each magnitude of change in frequency. Lines show the average number expected across 100 trials that randomized the sampling times among isolates. The final bar indicates counts of the number of SNPs with a predicted change >0.9 over time.

**Figure S5**. Predicted changes in the frequency of indel variants. Indels were filtered as described in the materials and methods, then binomial linear models were used to predict the changes in frequency over time. Lines show the predicted frequency of allele 1 versus allele 0 (where 0 is the allele of the designated reference genome for each species). Red lines highlight indels with >0.9 change in allele frequency during the time window sampled for each species. Note that each line can represent multiple overlapping indels showing the same pattern of predicted allele frequencies.

**Figure S6**. Variability in A) dN/dS ratios and B) pairwise amino acid divergence among samples of each species. Note *V. nonalfalfae* includes 2 *V. albo-atrum* samples.

FIGURE S1

FIGURE S2

FIGURE S3

FIGURE S4

FIGURE S5

FIGURE S6
